# A New Lunar Dome Detection Method Based on Improved YOLOv7

**DOI:** 10.3390/s23198304

**Published:** 2023-10-08

**Authors:** Yunxiang Tian, Xiaolin Tian

**Affiliations:** School of Computer Science and Engineering, Faculty of Innovation Engineering, Macau University of Science and Technology, Avenida Wai Long, Taipa 999078, Macau

**Keywords:** lunar dome, DEM data, attention mechanism, YOLO network

## Abstract

Volcanism is an important geological evolutionary process on the Moon. The study of lunar volcanic features is of great significance and value to understanding the geological evolution of the Moon better. Lunar domes are one of the essential volcanic features of the Moon. However, the existing lunar dome detection methods are still traditional manual or semiautomatic identification approaches that require extensive prior knowledge and have a complex identification process. Therefore, this paper proposes an automatic detection method based on improved YOLOv7 for lunar dome detection. First, a new lunar dome dataset was created by digital elevation model (DEM) data, and the effective squeeze and excitation (ESE) attention mechanism module was added to the backbone and neck sections to reduce information loss in the feature map and enhance network expressiveness. Then, a new SPPCSPC-RFE module was proposed by adding the receptive field enhancement (RFE) module into the neck section, which can adapt to dome feature maps of different shapes and sizes. Finally, the bounding box regression loss function complete IOU (CIOU) was replaced by wise IOU (WIOU). The WIOU loss function improved the model’s performance for the dome detection effect. Furthermore, this study combined several data enhancement strategies to improve the robustness of the network. To evaluate the performance of the proposed model, we conducted several experiments using the dome dataset developed in this study. The experimental results indicate that the improved method outperforms related methods with a mean average precision (mAP@0.5) value of 88.7%, precision (P) value of 85.6%, and recall (R) value of 86.4%. This study provides an effective solution for lunar dome detection.

## 1. Introduction

Volcanism is one of the main geological effects of the Moon, and the study of lunar volcanism can help analyze the composition of lunar soil and play an important role in understanding the process of lunar geological evolution. Domes are one of the typical volcanic geomorphic features produced by lunar volcanism on the lunar surface and are essential objects for studying the thermal evolutionary history of the Moon. Lunar domes often appear in clusters on the lunar surface, usually manifesting as an irregular round, convex shape, with low slope, low height, and a diameter of less than 30 km [1]. The origin of domes has been a controversial research issue for a long time. Early theories focusing on large-scale degassing suggested that gases beneath the surface layer caused the surface to swell upwards to form a dome structure [2,3]. However, a sufficient accumulation of gas beneath the surface to produce a dome several kilometers in diameter seems difficult to explain. Salisbury proposed a mineral-phase-change expansion hypothesis in 1961 [4]. Studies show that when serpentinization of olivine occurs below 500 °C, its volume increases by 25%. This seems to explain the reason for the formation of domes. Most scholars now believe that the domes were produced by lunar volcanism and formed in the late phase of lunar volcanism [1,5,6]. Depending on the different geological movements of lunar volcanoes, domes can be formed in two main ways. The first type is effusive domes, formed during the later stages of lunar volcanism when the lava extrusion rate decreases and the eruption temperature is relatively low. These domes usually have a summit crater pit that is a channel for lava outflow. The features of effusive domes are similar to those of small terrestrial lava shields. A further type of lunar dome is an intrusive dome, formed by subsurface intrusions of lava rather than lava effusion [1,7]. These domes usually do not have a summit crater pit but have an irregular outline [7]. Most of the dome formation is associated with the surrounding lava plains of roughly the same age. Large shield volcanoes like those on Earth do not occur on the Moon. This may be due to the low viscosity of lunar lava, the high eruption rate, and the lack of sustained eruptions from a single source [1,7,8].

Early dome classification and identification studies relied on telescopic observations and were recorded in several catalogs [1,9,10]. Smith proposed four criteria for dome identification and identified over 300 domes using lunar orbiter photographs in 1973. The morphological features of lunar domes are similar to those of terrestrial volcanic domes [5]. Head and Gifford classified more than 200 domes into seven categories based on morphological features [1]. These domes range from 3 to 17 km in diameter and several hundred meters in height and are mainly located in the Cauchy area, Hortensius area, and Marius Hills. Weitz and Head analyzed the titanium content of the domes in the Marius Hills complex with Clementine data [6]. The dome spectrum of the Marius Hills is similar to that of the mare plains, supporting the same compositions. The domes in the northern Oceanus Procellarum have redder and brighter soils than the adjacent highland materials representing the highlands. Wöhler et al. [7] classified lunar domes into four categories based on spectral features and morphological parameters using the principal component analysis method. Class A domes have a slope of less than 1°, are 5 to 13 km in diameter, and have a high TiO_2_ content. Class B domes consist of moderate TiO_2_ content and can be subdivided into two subclasses, depending on the duration of the lava eruption. Class B1 domes have a slope greater than 2° and are larger in volume, while Class B2 domes have a slope less than 2° and are relatively small. Domes with relatively low TiO_2_ content in class C domes are referred to as class C1, with domes ranging from 13 to 20 km in diameter and slopes of 0.6° to 1.8°. The class C2 TiO_2_ content is relatively high, the dome slope is less than 2.5°, and the diameter is 8 to 17 km. Class D is complex, with larger volumes, diameters, and slopes of 1.3° to 1.5°. Wöhler et al. [11] added a new class E based on the dome classification scheme in 2006 [7]. Class E domes are less than 6 km in diameter and can be divided into two subclasses based on slope: class E1 with slopes greater than 2° and class E2 with slopes less than 2°. For the completeness of the classification scheme, Class G was proposed for highland domes, corresponding to Class 6 in the Head and Gifford classification scheme [1]. Chen et al. used an unsupervised learning approach to cluster domes in the Gardner region and classified 54 domes into four classes [12]. By clustering five morphological features and two elemental features, the domes’ diameter, area, volume, and slope were derived as the optimal combination of parameters. DC1 domes are relatively small, steep, and formed by lavas with high viscosities and low eruption rates. The DC2 to DC4 domes are relatively large and smooth, forming lavas with low viscosities and high eruption rates [12].

Lawrence identified over 150 volcanic domes and 90 volcanic cones in the Marius Hills. This confirms that the Marius Hills dome is composed of blocky lava flows, and different morphological features of the domes and cones represent other eruptive conditions [13]. Liu established a contour-based dome identification method. Domes are identified by using contours to form a closed curve in the dome image, and then impact craters and irregular closed curves are removed [14]. Arya et al. identified 106 domes/cones in the Marius Hills and analyzed their morphological parameters. This suggests that wrinkle ridges are the oldest, rills are the youngest, and domes fall between them in age in the crater counting method [15]. Anto et al. presented a k-means clustering approach to identify domes based on digital terrain model data [16]. This method does not work well when the elevation value of the dome boundary is close to the elevation value of the lunar surface. By manual observation, Qiao et al. identified 283 domes in the mare Tranquillitatis [17]. The median diameter of these domes is 5.6 km, and the height is 68 m, of which 74% have summit pits. Using multisource remote sensing data, Wan identified 360 volcanic cones and 22 domes in the Marius Hills. Based on morphological characteristics, these volcanic cones were classified into five types: C-shaped, elliptical, elongated, irregular, and rounded [18]. Compared with terrestrial cones, the Marius Hills cones show larger basal widths and smaller height/width ratios related to multiple geological factors. Previous lunar dome identification studies have provided us with a comprehensive understanding of lunar domes’ morphological characteristics and spatial distribution. However, these are manual or semiautomatic methods that are complex workflows requiring much prior knowledge.

In recent years, most deep learning algorithms have achieved satisfactory results in object detection tasks [19,20,21,22,23,24]. These models can be categorized into one-stage methods and two-stage methods based on network architecture. The two-stage approach provides significant improvements in object detection performance, such as faster R-CNN, mask R-CNN, and Cascade R-CNN. In comparison, the one-stage approach has an advantage in terms of speed, such as YOLOv3 [23], and single-shot multibox detector (SSD) [20]. At present, the YOLO series methods have achieved great success in object detection. Zhu et al. integrated transformer block and convolutional block attention modules (CBAM) to improve the YOLOv5 model, referred to as TPH-YOLOv5 [25]. Arunabha et al. [26] proposed a high-performance road damage detection model, DenseSPH-YOLOv5, combining CBAM and swin-transformer prediction heads. Chen et al. presented a Citrus-YOLOv7 model based on YOLOv7 by adding a CBAM module and lightweight convolution [27]. The method has improved detection speed and can be used for the object detection of citrus fruits in a natural orchard environment. The CEAM-YOLOv7 [28] model using channel extension and attention mechanism techniques was proposed by Liu et al. This lightweight model leads to a significant increase in detection speed, but the detection accuracy still needs to be improved. Despite achieving satisfactory results, algorithms for lunar remote sensing images require improvement due to their complex backgrounds and similarities. Therefore, an automatic detection method based on improved YOLOv7 is proposed in this study. The main contributions of this study are summarized in three parts as follows:

1. We performed a rendering operation on the DEM images based on the elevation values to enhance the contrast of the domes. The ESE attention module was added to the backbone and neck sections to reduce information loss in the dome feature map.
2. We proposed a new SPPCSPC-RFE module by adding the RFE module into the neck section to adapt dome feature maps of different shapes and sizes.
3. The bounding box regression loss function CIOU was replaced by WIOU to improve the performance of the model in dome detection.

The rest of this paper is organized as follows. Section 2 introduces the data used in this study and proposes an improved YOLOv7 model. Section 3 shows the experimental results. Section 4 is the conclusion of this paper.

## 2. Dataset and Methods

### 2.1. Dataset

**Figure 1:**
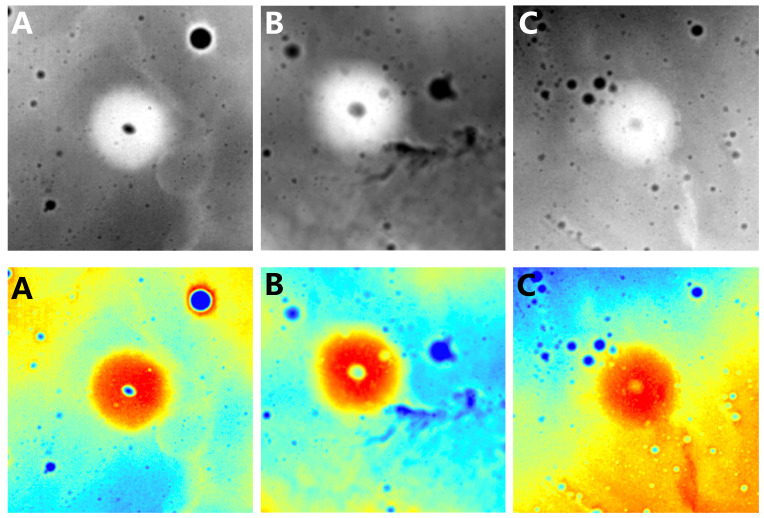
Three representative lunar domes (**A**–**C**) are shown with original DEM images and colored DEM images.

The main features of lunar domes are typically smooth, low-sloped convex profiles, low height, and diameter less than a few tens of kilometers [1,29]. These unique topographical features make lunar domes possible to observe only in images of low sunlight [17,30,31]. However, DEM data are unaffected by solar illumination conditions and can represent dome morphological features such as the height of the domes. As shown in Figure 1, lunar domes are usually represented as circular structures higher than the surrounding area in DEM data. For these reasons, we chose DEM data proposed by Barker et al. in 2016 [32] for analysis and experimentation in this study.

We reinvestigated the entire lunar domes, based on the previous identification results [1,6,7,12,13,15,16,17] and the two dome catalogs [33,34]. The study obtained a total of 771 domes, of which 597 were confirmed domes and another 174 were suspected domes. Cropping the entire lunar DEM image based on latitude and longitude coordinates and diameter information, a total of 680 dome images were obtained. Based on the results of the expert confirmation, the 680 images were divided into two parts, with 519 images containing 597 confirmed domes and another 161 images containing 174 suspected domes. To obtain reasonable experimental results, 519 confirmed dome images were used as a training set, and 161 suspected dome images were used as a test set. At a ratio of 4:1, 519 images were further divided into 104 images for the validation set and 415 images for the training set. Several data enhancement strategies were used in this study to improve the model’s robustness and avoid overfitting. First, a rendering operation was used for each image individually rather than for the entire lunar image to represent the morphological features of the domes better. As shown in Figure 1, we used red to represent relatively high terrain and blue to represent relatively low terrain. This rendering operation makes distinguishing between impact craters and domes easy and shows the dome boundaries more effectively. In our dataset, domes are usually shown as red circular shapes, while impact craters are shown as blue circular shapes. Then, other data enhancement strategies were used, including random scaling, flipping, brightness enhancement, contrast enhancement, and color enhancement. The training set was increased to 1500 images through the above data-enhanced operations.

### 2.2. YOLOv7 Network Structure

**Figure 2:**
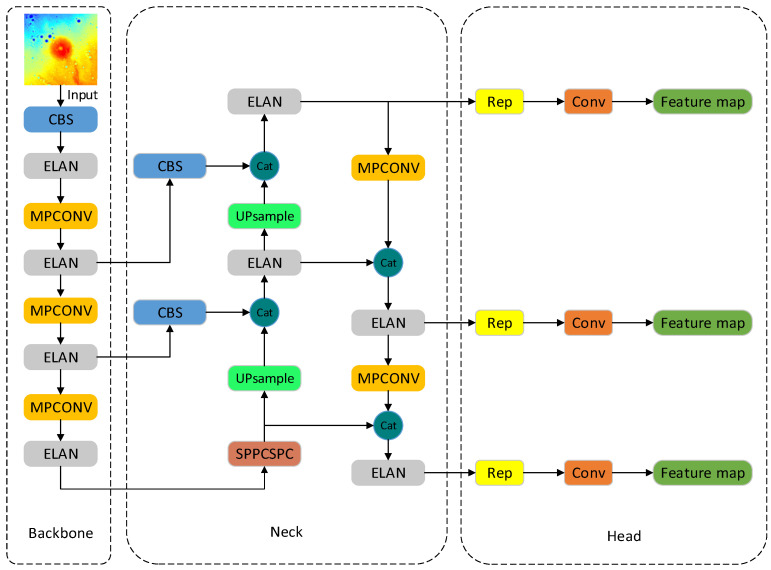
Structure of the YOLOv7 module.

The YOLOv7 [35] network is one of the more advanced target detection algorithms, and its detection speed and accuracy exceed those of most known target detection algorithms. The YOLOv7 network consists of four sections: input, backbone, neck, and head, as shown in Figure 2. The function of the input module is to scale the input image to meet the input size requirements of the backbone network. The backbone network comprises CBS convolution layers, efficient layer aggregation network (ELAN) convolution layers, and max-pooling convolution (MPconv) layers. The role of the CBS module is to perform feature extraction and channel transformation. The ELAN module is an aggregation network structure that enriches the feature information extracted from the backbone structure by changing the feature extraction paths and improving the robustness of the model. The MPconv adds a max-pooling layer on top of the CBS layer to form the upper and lower branches and finally uses the concat operation to fuse the features extracted from the two branches, improving the network’s feature extraction capability. The neck module is composed of a path aggregation feature pyramid network (PAFPN) structure. The PAFPN makes the network suitable for multisized inputs and fully integrates the underlying precise positional information with the high-level abstract semantic information to improve the localization accuracy of the model further. In the detection head module, the YOLOv7 network utilizes three target sizes: large, medium, and small. Through the RepVGG (REP) block for the PAFPN, outputs of different sizes of features are adjusted in terms of the number of channels, and combined with 1 × 1 convolution, the prediction of the position, confidence, and category of the target object is derived.

### 2.3. Improvement to the YOLOv7 Network

#### 2.3.1. ESE Attention Module

**Figure 3:**
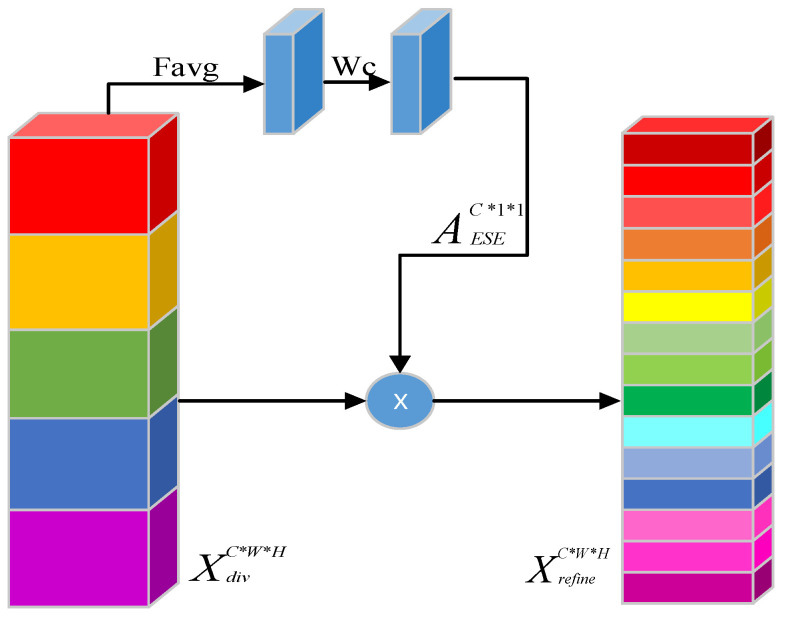
Structure of the ESE attention module.

Researchers have explored the relationships between channels within the network in developing deep learning networks in recent years. Hu et al. [36] constructed a squeeze-and-excitation (SE) block by explicitly modelling the interdependencies between channels and adaptively recalibrating the feature responses in the channel directions. The process of an SE block is divided into two steps: squeeze and excitation. Squeeze performs global average pooling on the previous feature map layer to obtain the global compressed feature vectors of the current feature map. Excitation obtains the weights of each channel in the feature map through two fully connected (FC) layers and uses the weighted feature map as an input to the next layer of the network. The SE module improves convolutional neural network performance, but reducing channel size can cause information loss. When the first FC layer decreases the number of input channels, the second layer needs to increase the number of channels back to the original amount. However, this process results in loss of channel information. To address information loss, the ESE module was proposed [37]. It is based on the SE module but uses only one FC layer, reducing channel information loss and improving network performance. In this paper, we added several ESE attention modules into the YOLOv7 network. The ESE Attention module optimizes the extracted features, considering network depth, and detection speed to improve dome detection accuracy with minimal increase in parameters. The ESE module is defined as follows:

(1) $$F_{\mathrm{gap}}\left(X\right)=\frac{1}{\mathrm{WH}}\sum_{i,j=1}^{W,H}X_{i,j}$$

(2) $$A_{\mathrm{ESE}}\left(X_{\mathrm{div}}\right)=\sigma (W_{C}(F_{\mathrm{gap}}\left(X_{\mathrm{div}}\right))$$

(3) $$X_{\mathrm{refine}}=A_{\mathrm{ESE}}\left(X_{\mathrm{div}}\right)⊗X_{\mathrm{div}}$$

where $F_{\mathrm{gap}}\left(X\right)$ is global average pooling, σ indicates the sigmoid function, $W_{C}$ is the weights of the FC layers, $X_{\mathrm{div}}$ is the input feature map, $X_{\mathrm{refine}}$ is the refined feature map, and ⊗ indicates elementwise multiplication. The ESE module flowchart is shown in Figure 3. The $A_{\mathrm{ESE}}$ operation is applied to the feature map and leads to diversified feature information, which retains channel information and improves model performance.

#### 2.3.2. SPPCSPC-RFE Module

**Figure 4:**
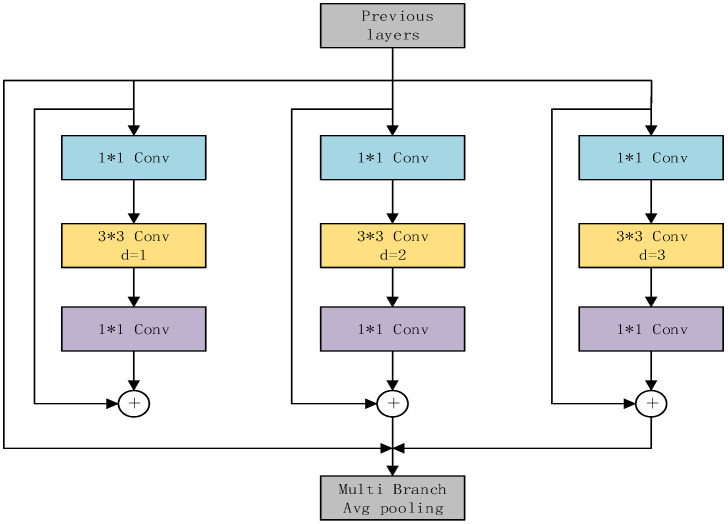
Structure of the RFE module.

In many scenarios, the lunar surface exhibits domes of varying size and structure, creating difficulties for detection. Although pyramids can solve the multiscale problem, for smaller domes, there is still the problem of information loss after multiple convolution operations. Different receptive fields indicate the ability to capture long-range dependency [38]. Increasing the receptive fields of the feature map can aid in detecting smaller domes. The RFE module can increase the receptive field and improve multi-scale feature fusion. The RFE module adequately captures the information in the feature map using dilated convolution, which uses four different branches to capture multiscale information. Each branch shares common weights, but their receptive fields are independent. The advantage of this approach is that the model parameters can be reduced while reducing the risk of overfitting, and the features can be fully utilized. As shown in Figure 4, the RFE module can be divided into two parts: the multibranch structure and the gathering and weighting layer. The multibranch structure uses different convolution rates and residual connections to prevent the problem of gradient disappearance during training. The gathering and weighting layer collects information from other branches and weights each branch [38].

**Figure 5:**
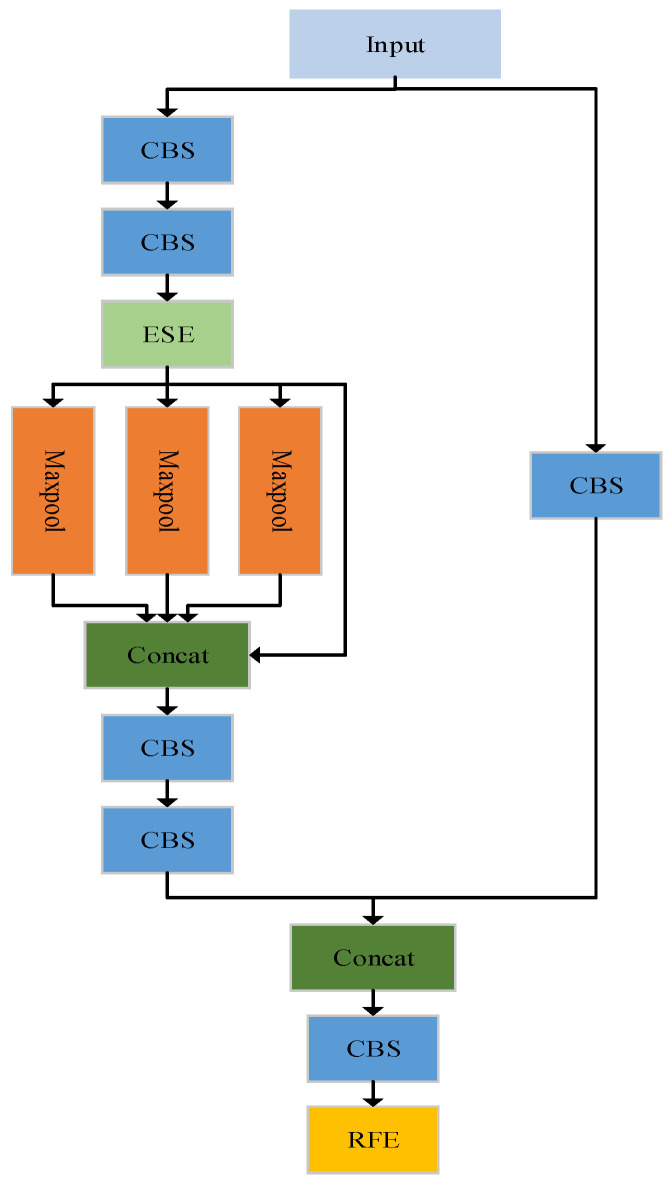
SPPCSPC-RFE network structure.

In the neck section, the role of the SPPCSPC module is to improve the efficiency and accuracy of feature extraction. The SPP layer can capture information about objects of different scales and proportions, the CSP connection can enhance the expressiveness and stability of the features, and the conv layer can further extract features. A new SPPCSPC-RFE module is proposed in which the ESE and RFE modules are embedded in the SPPCSPC module to extract features better while reducing information loss. The new network structure is shown in Figure 5. The CBS module is replaced by the ESE module before the max-pooling layer to reduce the loss of feature information. The RFE module is added to the end of the SPPCSPC module to retain more feature map information and improve the network’s performance.

#### 2.3.3. Bounding Box Regression Loss Function

The bounding box regression loss function used in the original YOLOv7 network is CIOU. Although CIOU achieved good results, the calculation of aspect ratios used relative values and needed to consider the balance of difficult and easy samples. Therefore, we replaced the original bounding box regression loss function CIOU with the WIOU [39] based on a dynamic nonmonotonic focusing mechanism in this study. The WIOU loss function reduces the competitiveness of high-quality anchor boxes and avoids the impact of low-quality examples. It allows WIOU to focus on ordinary quality anchor boxes and improve the overall performance of the network. The formula is as follows:

(4) $$L_{\mathrm{WIOU}}=\frac{\beta }{{\delta \alpha }^{\beta -\delta }}\exp(\frac{{\left(x-x_{\mathrm{gt}}\right)}^{2}+{\left(y-y_{\mathrm{gt}}\right)}^{2}}{{\left(W_{g}^{2}+H_{g}^{2}\right)}^{*}})L_{\mathrm{IOU}}$$

where $L_{\mathrm{IOU}}$ is the value of the loss function, exp is an exponential function, δ and α are hyperparameters, β is the outlier degree of the anchor box, x and y are the coordinates of the center point of the prediction box, $x_{\mathrm{gt}}$ and $y_{\mathrm{gt}}$ are the coordinates of the center point of the ground truth box, $W_{g}$ and $H_{g}$ are the sizes of the smallest enclosing box, and $L_{\mathrm{IOU}}$ is the intersection ratio of the overlapping areas of the predicted and ground truth boxes.

**Figure 6:**
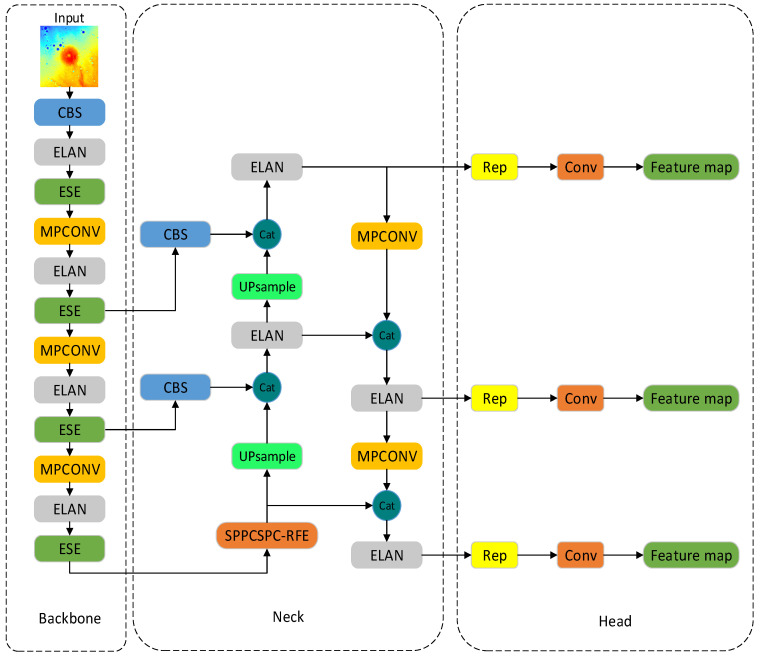
Structure of the improved YOLOv7 module.

In summary, the improved YOLOv7 model is shown in Figure 6.

## 3. Experimental Results

### 3.1. Experimental Environment Configuration

The software environment used for the experiments in this study was the Ubuntu system, Python 3.8, CUDA11.3, and PyTorch 1.11. The hardware environment was an AMD EPYC 7543 CPU with 32 G RAM and an NVIDIA RTX A5000 GPU (24 G). In the experiments, the image size in training and testing was set to 640 × 640, and the batch size was 24. The optimizer used SGD with a learning rate of 0.02.

### 3.2. Evaluation Metrics

To objectively evaluate the performance of the model in lunar dome detection, the parameters of floating point operations per second (FLOPS), recall (R), precision (P), and mean average precision (mAP) evaluation metrics were used in this study. The P and R of the dome detection metrics can be calculated using the following formulas:

(5) $$P=\frac{\mathrm{TP}}{\mathrm{TP}+\mathrm{FP}}$$

(6) $$R=\frac{\mathrm{TP}}{\mathrm{TP}+\mathrm{FN}}$$

In the formulas, TP means the number of true positive samples, FP means the number of false positive samples, and FN represents the number of false negative samples. AP represents the average precision. The AP value is the area enclosed by the curve plotted by P and R between 0 and 1 and the axis. The mAP value represents the mean average precision, where C denotes the number of detection target categories and i is the category index. mAP@0.5 represents the mean average precision when the intersection ratio threshold is 0.5. The formulas are shown as below:

(7) $$\mathrm{AP}=\int_{0}^{1}P\left(R\right)$$

(8) $$\mathrm{mAP}=\frac{1}{C}\overset{C}{\underset{i=1}{\sum }}{\mathrm{AP}}_{i}$$

(9) $$\mathrm{mAP}@0.5=\frac{1}{C}\overset{C}{\underset{i=1}{\sum }}\mathrm{AP}@0.5_{i}$$

### 3.3. Results

**Figure 7:**
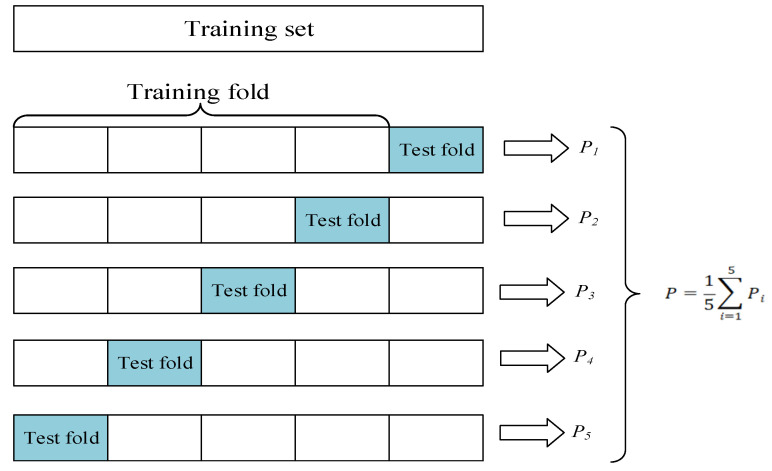
Flow chart of five-fold cross-validation method for precision.

In our experiments, we utilized a k-fold cross-validation approach to obtain a more accurate model assessment. This method avoids problems caused by irrational division of the dataset. In this study, we chose k equal to five. As shown in Figure 7, the training set was divided into five parts. In the training process, one part was used as the validation set, and the others were used as the training set. Finally, the average of five precision values was calculated as the actual precision rate of the model. To verify the effectiveness of the improvements of the YOLOv7 model proposed in this paper, we designed multigroup ablation experiments on our dataset, and the results of the ablation experiments are shown in Table 1, where “√” indicates that the module is used in the current experiment.

As shown in Table 1, the mAP@0.5, P, and R of the original YOLOv7 model without the improvement module reached 83.6%, 82.7%, and 77.0%, respectively. The second set of experiments added a data enhancement strategy to the original YOLOv7 model, increasing the mAP@0.5, P and R by 1.3%, 1.9%, and 4.9%, respectively. The ESE attention module was added to the group 3 experiment based on the second set of experiments. It made the model more focused on the detection object and filtered out irrelevant background information, which improved the mAP@0.5 by 1.6% and decreased R by 4.3%. Then, the RFE module was added to the fourth set of experiments, which increased the mAP@0.5 by 1.2% and R by 6.3%. Furthermore, the CIOU loss function was replaced by the WIOU loss function in group 5. The best performance of the model was obtained, and the mAP@0.5, P, and R of the improved model reached 88.7%, 85.6%, and 86.4%, respectively. At the same time, when new modules were added, the number of parameters of the model increased slightly within a reasonable range.

Comparative experiments were conducted in the same experimental environment to demonstrate the excellent identification ability of the proposed method in this paper. The study compared the performance of the following models: SSD [20], YOLOv3 [23], Faster-RCNN [19], YOLOv5 [40], TPH-YOLOv5 [25], YOLOv7 [35], Citrus-YOLOv7 [27], and our proposed model.

**Figure 8:**
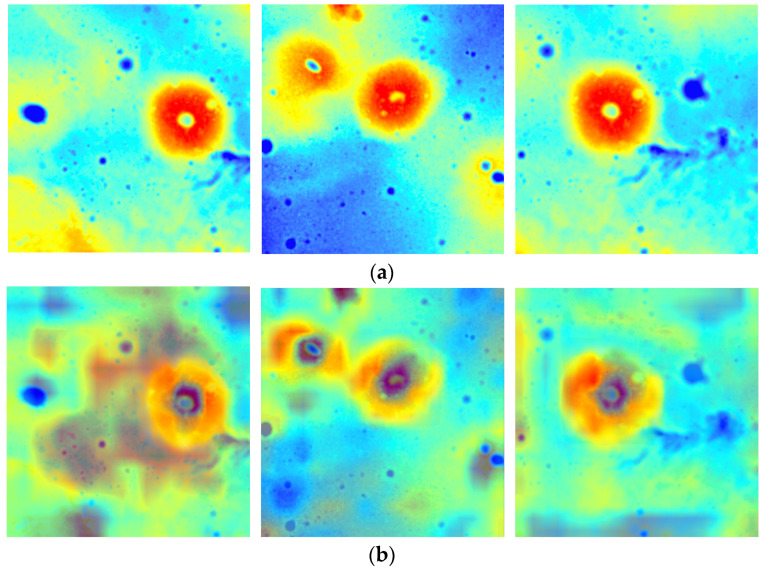

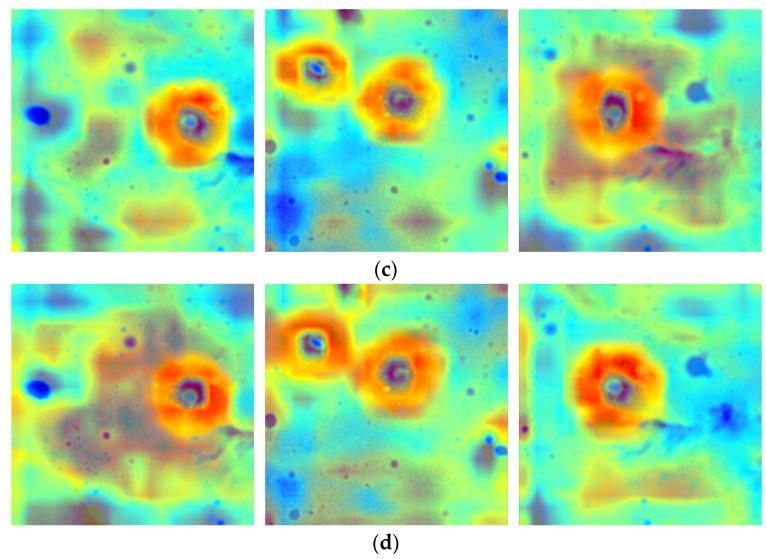
Heat maps of different models. (**a**) Colored DEM images, (**b**) Original YOLOv7, (**c**) Citrus-YOLOv7, (**d**) ours.

**Figure 9:**
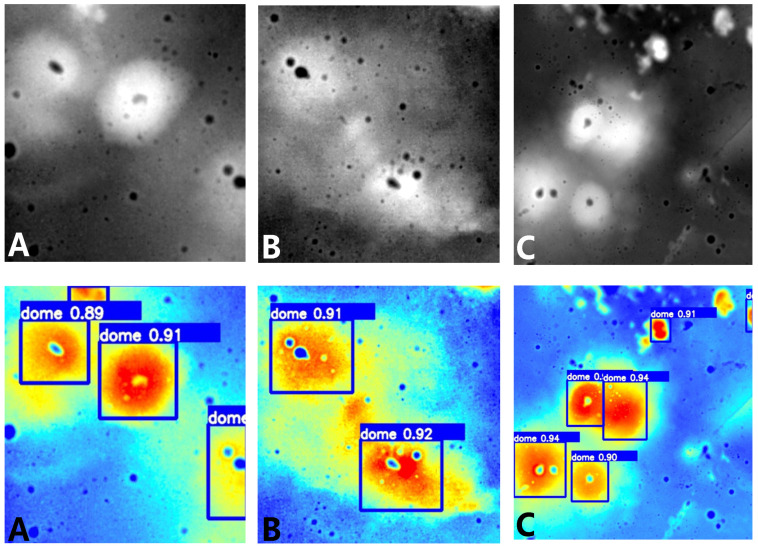
Examples of dome detection results (**A**–**C**) are shown with original DEM images and our method.

Table 2 shows that the proposed method reached the highest MAP@0.5 value of 88.7%, P value of 85.6%, and R value of 86.4%. This indicates that our improved model has the best performance in lunar dome detection. Although the SSD and YOLOv3 models have lower complexity, the values of MAP@0.5, P, and R were relatively low. Compared with the YOLOv5 model, the proposed model increased the mAP@0.5, P, and R by 5.5%, 0.8%, and 8.3% respectively. At the same time, the parameters of the model were reduced somewhat. Although the TPH-YOLOv5 method reached the highest MAP@0.5:0.9 value of 52.1%, the values of MAP@0.5 and R are relatively low. The proposed model improves the mAP@0.5, P, and R by 3.8%, 1.0%, and 4.5%, respectively, compared to the original YOLOv7 model. The improved YOLOv7 model slightly increases the number of model parameters, which is within a reasonable range. In addition, our proposed method outperforms TPH-YOLOv5 and Citrus-YOLOv7 models while having similar complexity. To better demonstrate the effectiveness of our method in distinguishing foreground and background in dome images, we present Figure 8 which visualizes the attention regions for each model. The network model pays more attention to the red area, which has a darker color. As seen from the heat maps in Figure 8b,c, the network attention of both models could not be focused on the entire dome region. The heat map Figure 8d demonstrates our model’s ability to accurately identify the contour lines of the dome while maintaining attention on overall characteristics of the target. Examples of dome detection results are shown in Figure 9. In summary, the improved model provides high detection performance in complex lunar terrain.

## 4. Conclusions

This paper proposes an automated method for detecting lunar domes using an im-proved YOLOv7 network. The method introduces a novel approach for recognizing complex lunar dome images. First, a new lunar dome dataset was created by DEM data, and an ESE attention module was added to the backbone and neck sections to reduce information loss in the feature map and enhance network expressiveness. Then, the RFE module was embedded into the neck section, which can adapt to dome feature maps of different shapes and sizes. Finally, the bounding box regression loss function CIOU was replaced by WIOU. In addition, several data enhancement strategies were applied to the dataset. The results of ablation experiments show that the mAP@0.5 value and R value of the improved algorithm reached 88.7% and 86.4%, respectively, which were increased by 5.1% and 9.4% compared with the original YOLOv7 algorithm. The comparison experiments show that the proposed algorithm has better results than state-of-the-art detection networks. Although our proposed method achieves satisfactory results, this approach results in a slight increase in the number of model parameters. When the elevation value of the dome is close to the lunar surface, it becomes challenging to differentiate the dome boundary from the surface using our method. In the future, we will use context learning to fully utilize the target information in the images and combine lunar data from different sources to improve the detection performance of the model. Meanwhile, we will explore ways to decrease the number of model parameters to further enhance its performance.

## Figures and Tables

**Table 1:** Ablation experiment results.

Model	Group	Data Enhancement	ESE	RFE	WIOU	MAP@0.5 (%)	MAP@0.5:0.9 (%)	P (%)	R (%)	Parameters
	1					83.6	44.9	82.7	77.0	37.62
	2	√				84.9	48.7	84.6	81.9	37.62
YOLOv7	3	√	√			86.5	50.7	87.5	77.6	39.1
	4	√	√	√		87.7	48.1	87.4	83.9	39.3
	5	√	√	√	√	88.7	52.0	85.6	86.4	39.9

**Table 2:** Comparison of experimental results with other network models.

Method	MAP@0.5 (%)	MAP@0.5:0.9 (%)	P (%)	R (%)	Parameters	GFLOPS
SSD	83.1	45.3	81.9	59.5	24.39	34.27
YOLOv3	82.2	42.3	80.2	51.6	21.2	49.0
Faster-RCNN	85.0	49.8	85.0	59.6	41.12	91.0
YOLOv5	83.2	51.8	84.8	78.1	43.97	107.6
TPH-YOLOv5	84.6	52.1	84.5	81.6	44.0	108.2
YOLOv7	84.9	48.7	84.6	81.9	37.62	106.5
Citrus-YOLOv7	85.6	47.5	82.5	84.3	26.44	76.4
Ours	88.7	52.0	85.6	86.4	39.9	107.3

## Data Availability

Not applicable.
